# Letter commenting on ‘The role of experience level in radiographic evaluation of femoroacetabular impingement and acetabular dysplasia’ by Patrick C. Schottel *et al.*

**DOI:** 10.1093/jhps/hnv031

**Published:** 2015-05-04

**Authors:** Thomas Sampson

**Affiliations:** Director Hip Arthroscopy Post Street Surgery, Orthopaedic Consultant Veterans Administration and Faculty, University of California, San Francisco

Patrick C. Schottel *et al.* in their study, ‘The role experience level in radiographic evaluation of femoroacetabular impingement and acetabular dysplasia,’ studied the interobserver and intraobserver reliability for the radiographic evaluation and diagnosis of femoroacetabular impingement (FAI) and acetabular dysplasia (AD) also known as developmental dyspalsia of the hip (DDH) between physicians with varying levels of experience, with a premise that there may be less concordance compared to other studies among only orthopaedic hip specialists. Additionally they assumed by earlier radiographic diagnoses of FAI and AD would lead to earlier hip preservation and reduce early osteoarthritis (OA). They hypothesize ‘FAI and AD often have overlapping symptoms, making an accurate diagnosis challenging. Therefore, radiographs remain the cornerstone in the evaluation, diagnosis and management of patients with these pre-arthritic hip condition,’ and ‘the ability to accurately and consistently interpret the radiographs in this particular patient population is of great importance.’ They used 14 parameters (6 objectives and 8 subjectives) to evaluate 55 hips hip radiographs with on follow-up 6 weeks later on 20 hips for a reliability test, with blinded radiographic reviews of all 55 patients by an attending orthopaedic hip surgeon, an attending musculoskeletal radiologist, an orthopaedic sports fellow and a 3-year orthopaedic surgery resident. They found ‘a relatively low level of interobserver and intraobserver reliability between readers, especially for subjective parameters. Thus, many of the standard radiographic measurements on anterior posterior (AP) pelvis and lateral hip views to diagnose FAI or AD were observed as not being reproducible,’ and found interobserver reliability to be highest among objective parameters such as the lateral center edge angle and alpha angle. Poor interobserver reliability values were observed with predominately subjective measures such as joint congruency, Tönnis grade and detection of herniation pits. The unique aspect of their study was that the amount of surgical hip experience increased the likelihood of radiographically making a pathologic diagnosis with their senior hip surgeon demonstrating the highest diagnostic sensitivity among all readers; however, he also demonstrated the lowest degree of diagnostic specificity with over reading normal radiograph as abnormal. They concluded a tendency to over diagnose radiographs can explain the increasing incidence of hip arthroscopy procedures. While this study uses 14 parameters to radiographically diagnose FAI and AD, most astute clinicians base their diagnosis on a sound history and an accurate physical exam the radiographs as an adjunct to their clinical impression. We know from other studies that radiographic FAI may not necessarily be symptomatic [1, 2], just as magnetic resonance imaging evidence of labral tears is present in 73% of asymptomatic hips [3]. I estimate that most hip specialists use only 4–5 of the 14 parameters along with their clinical impression to accurately diagnose FAI and AD, and the over-read by the attending hip specialist came from his experience on treating patients with normal radiographs and severe pathology seen at arthroscopy as has been with my personal experience. Their conclusion that objective radiographic measurements such as lateral center edge angle and alpha angle had stronger interobserver and intraobserver reliability than more subjective measurements such as Tönnis grade may be that those are more widely used, however, I am not in agreement that subjective radiographic parameters need to be redefined and objective parameters need to be further developed to improve the reliability for accurately diagnosing FAI or AD. Those are already well defined when combining the data with the clinical impression.
